# Barriers against and strategies for malaria control during the COVID-19 pandemic in low- and middle-income countries: a systematic review

**DOI:** 10.1186/s12936-023-04452-2

**Published:** 2023-02-03

**Authors:** Jiwook Park, Seungwoo Kang, Dayoung Seok, Yae Jee Baek, Se Young An, Junga Lee, Alina Jun, Sun-Young Kim

**Affiliations:** 1 Graduate School of Public Health, Seoul National University, 1 Gwanak-Ro, Gwanak-Gu, Seoul, 08826 South Korea; 2 Division of Infectious Disease, Department of Internal Medicine, Soonchunhyang University Seoul Hospital, Seoul, Korea; 3 Institute of Health and Environment, Seoul National University, Seoul, Korea

**Keywords:** Malaria, COVID-19, Pandemic, Barriers, Malaria strategies, Low- and middle-income countries (LMICs), Systematic review

## Abstract

**Background:**

The COVID-19 pandemic has disrupted malaria control activities globally. Notably, high levels of excess malaria morbidity and mortality in low- and middle-income countries (LMICs) were reported. Although it is crucial to systematically understand the main causes of the disruption to malaria control and synthesize strategies to prepare for future pandemics, such studies are scarce. Therefore, this study aims to better identify barriers against and strategies for malaria control.

**Methods:**

Following the PRISMA guidelines and through searches of electronic databases and Google Scholar, a systematic literature review was conducted to identify studies pertaining to malaria control published between January 2020 and December 2021. Only studies that discussed reported barriers and/or strategies related to malaria were included for the review. The Mixed Methods Quality Appraisal Tool (MMAT) and the Authority, Accuracy, Coverage, Objectivity, Date and Significance (AACODS) checklist were used for quality appraisal. Key information such as literature type, study design, setting and population, interventions, outcomes, barriers, and strategies were extracted. With an existing framework of four dimensions (accessibility, affordability, availability, and acceptability) further subdivided by the supply and demand sides, this study synthesized information on barriers and strategies related to malaria control and further categorized the strategies based on the time frame.

**Results:**

From the 30 selected studies, 27 barriers and 39 strategies were identified. The lockdown measures, which mainly threatened geographic accessibility and availability of malaria control services, were identified to be the main barrier hindering effective mobilization of community health workers and resources. Among the identified strategies, clear risk communication strategies would alleviate psychosocial barriers, which challenged acceptability. Some strategies that cross-cut points across all four dimensions would, require systems-level integration to enhance availability and affordability of malaria control. The strategies were distinguished between short-term, for instant response, and mid to long-term for future readiness.

**Conclusions:**

The pandemic resulted in complex barriers to malaria control, particularly imposing a double burden on LMICs. Identifying strategies to overcome said barriers provides useful insights in the decision-making processes for the current and future pandemic. Cross-cutting strategies that integrate all dimensions need to be considered. Health system strengthening and resilience strategy appropriate for country-specific context is fundamental.

**Supplementary Information:**

The online version contains supplementary material available at 10.1186/s12936-023-04452-2.

## Background

Malaria is an acute febrile illness caused by a parasite transmitted through the bites of infected mosquitoes. It has been a deadly threat in most countries for centuries. Malaria control, which was abandoned and considered to be a failure in the 1960s, was recognized as a global health priority in the 2000s [1]. As malaria control was designated as one of the Millennium Development Goals, many countries have prioritized combating malaria as a public health issue. Many global health initiatives, such as the Global Fund or Roll Back Malaria, were established to fight malaria by collecting funds and scaling up malaria interventions. Many organizations and governments have seen substantial progress through well-implemented malaria prevention, treatment, and vector control [2]. Consequently, a considerable reduction in malaria morbidity and mortality has been observed. However, even though many malaria-endemic countries have achieved remarkable progress in malaria control, with some even succeeding in malaria elimination, malaria remains a major global public health concern [3].

The emergence and spread of the coronavirus disease 2019 (COVID-19) have adversely affected almost all countries. Not only has COVID-19 killed millions of people, but the pandemic’s impact has also gone beyond direct infections and death tolls. The coronavirus has forced countries into lockdown, ravaged economies, pushed millions into extreme poverty, and disrupted essential health services. Similarly, the COVID-19 pandemic has devastated malaria control programmes [4]. In other words, because of the emergence of the COVID-19 pandemic, malaria control has seen significant setbacks despite numerous global health initiatives [5]. The disrupted malaria control program’s consequences are further exacerbated in low- and middle- income countries (LMICs) because of vulnerable health systems and a larger burden imposed by malaria. The weak health system makes these countries more susceptible to the repercussions of the COVID-19 pandemic. Furthermore, in the early stages of the pandemic, off-label use of anti-malarial drugs was presumably considered to be effective in treating COVID-19, resulting in a shortage of the medications. LMICs experienced a similar situation back in 2014 and 2015: the Ebola virus disease (EVD) outbreaks in West Africa indirectly increased the mortality of endemic diseases such as malaria. During the EVD outbreaks, malaria deaths significantly increased and exceeded those from the Ebola virus [6, 7].

Scholars adopted mathematical modelling to predict the deaths or cases of malaria in the early stages of the COVID-19 pandemic [8, 9]. According to the World Malaria Report 2021, malaria cases and deaths in 2020 increased by 6% (245 million) and 12% (627,000), respectively, compared to the previous year, adversely affecting the target of the Global Technical Strategy for Malaria 2016–2030 (GTS) [10]. Some other studies have reported the impact of the COVID-19 pandemic on malaria control and identified the challenges contributing to the excess cases and deaths during the pandemic [11, 12]. However, there have been no attempts to systematically review the contexts of the barriers to malaria services in LMICs amidst the global pandemic and the nature of the strategies to overcome such barriers. Therefore, this study aims to identify barriers that disrupt malaria control activities in LMICs during the COVID-19 pandemic and strategies to move forward. In particular, this study builds upon the analytical framework that Jacobs et al. used to investigate obstacles in low-income Asian and African countries during the 2014–2015 EVD outbreak [13].

## Methods

### Search strategy

**Fig. 1:**
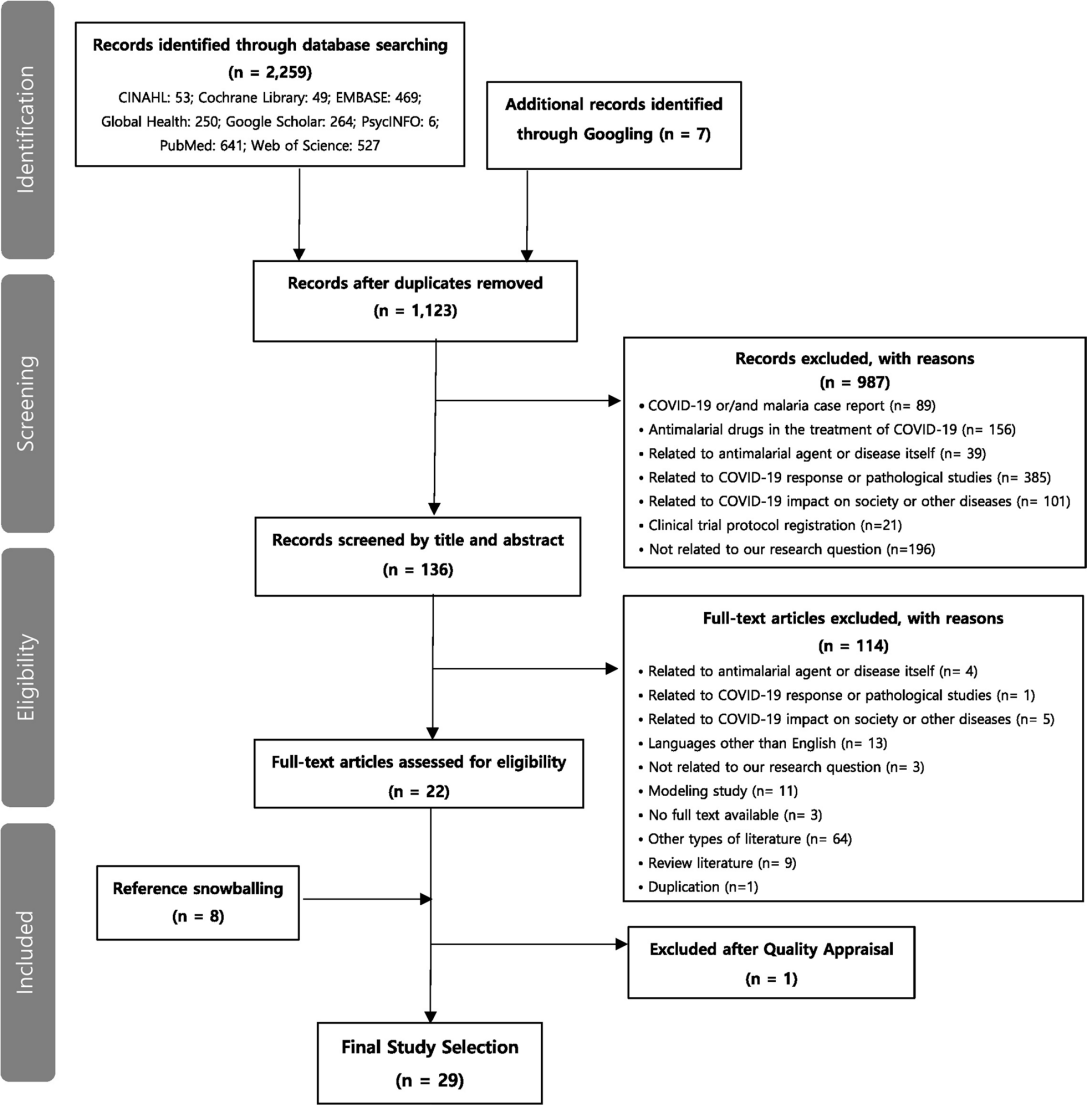
PRISMA flow chart of study selection

Following the PRISMA guidelines, a systematic literature review (Fig. 1) was conducted to identify studies on barriers against and strategies for malaria control during the COVID-19 pandemic in LMICs. The self-reported PRISMA checklist can be seen in Additional file 3, 4. Electronic databases including PubMed, Cochrane Library, Embase, CINAHL, Web of Science, PsycInfo, Global Health as well as Google Scholar were used to identify relevant literature. The search focused on studies published in the last two years from January 2020 to December 2021. Variants of the following search terms were used: malaria, COVID-19, and LMICs. The complete search terms are annexed [see Additional file 1]. Additionally, relevant grey literature (e.g., reports) were also obtained through additional searches. Lastly, the reference lists of selected literature were examined to identify any additional articles that fit the inclusion criteria.

### Eligibility criteria

Original research conducted in English that examine barriers to and strategies for control of malaria in LMICs in the era of the COVID–19 pandemic were included. The category of the countries was based on the World Bank List of Economies as of the 2022 fiscal year. In addition to peer-reviewed publications, limited types of grey literature (i.e., reports from the major malaria-related actors and preprints of journal articles) were included in the analysis as there was a limited number of published literature on hoarding during the COVID-19 pandemic. Similar search strategies were used to identify such grey literature. If the preprints were published prior to journal publication, the latest updates were reflected. According to the inclusion criteria, only literature released by December 2021 should have been included in the analyses. However, some articles that were published in 2022 (after their original inclusion into this review as preprints in 2021) were exceptionally included. Literature was excluded if the authors did not address either barriers or strategies in their own analysis or actual outcomes related to malaria control as in the cases of modelling studies or study protocols. In addition, case reports, inability to access full text, and grey literature (other than reports and preprints) were criteria by which literature was excluded from the analysis.

### Study selection

The results of the final search were collated using EndNote 20. The selection of literature was conducted in two stages after removing duplicates. At the first stage, the title and abstracts of the retrieved literature were screened. In the next stage, the literature that was considered potentially relevant to this study’s topic was assessed for eligibility and fully read. All processes were conducted independently by two randomly assigned reviewers, and any conflicts of inclusion were resolved through a team consensus.

### Assessment of methodological quality

The Mixed Methods Quality Appraisal Tool (MMAT) and the Authority, Accuracy, Coverage, Objectivity, Date and Significance (AACODS) checklist were used to explore the risk of bias at the individual study level. The articles, whether published in peer-reviewed journals or in progress as preprints, were assessed using the MMAT version 2018. The MMAT is a methodological quality appraisal tool designed for systematic reviews encompassing studies with various types of study designs [14]. One point was given for each of the criteria met and the maximum possible score for each article was seven. The AACODS checklist was used for grey literature as the MMAT methodology is not fit for grey literature [15]. A point was given for each of the six criteria and an overall score was calculated for each literature. Similarly, two researchers independently identified the study designs and scored the literature. Any conflicts were discussed and adjusted.

### Data extraction

Key information from eligible literature was extracted and summarized into a predetermined template, consisting of categories such as: literature type, study design, study setting, study population, interventions, outcomes, barriers to malaria control, and strategies. In this study, “barriers to malaria control” were defined as obstacles that restrain interventions for malaria prevention, diagnosis, and treatment that are planned or implemented during the COVID-19 pandemic in LMICs. Researchers extracted barriers from selected studies only when the barriers were identified by the literature under review, were explicitly mentioned and identified by the authors, all the while considering the context, analysis, and results of each study. The barrier extraction process excluded indirectly induced barriers that referred to other literature. The extracted barriers were thoroughly cross-checked and categorized by two independent reviewers. Disagreements were resolved through team discussion.

### Data synthesis

The study categorized the identified barriers to malaria control based on the analytical framework developed by Jacobs et al. [13] for assessing barriers to health services. The framework was built upon the study by Ensor & Cooper, which assessed barriers while taking into account the supply and demand sides as well as the four dimensions of Peters et al. [16, 17]: accessibility, affordability, availability, and acceptability. The descriptions of the four dimensions by Peters et al. [17] were modified for this study to better fit the context of malaria control during the pandemic. The strategies identified from the previous literature were also categorized using the same framework. The categorized barriers and strategies were divided into two subcategories: supply and demand sides. In addition, the strategies were further stratified into short-term, mid-term, and long-term based on the time by which the goals are likely to be achieved. In the subsequent analysis, short-term strategies are defined as “immediate responses which take place within days to weeks after disruptions.” Long-term responses are defined as “those that go on for years after disruptions.” Mid-term strategies are defined as “responses that take place at any timeframe between short- term and long-term.”

## Results

Searches through electronic databases and the Google Scholar search engine yielded 2,266 documents. A total of 1,123 studies were screened after removing duplicates. The process of title and abstract screening and full text review excluded 987 and 114 studies, respectively. Figure 1 shows detailed reasons for exclusion. Eight documents were added through reference snowballing. In the methodological quality assessment, one study was excluded as one of the MMAT screening questions were not satisfied. The result of the quality assessment is summarized in the annex [see Additional file 2].

A total of 29 studies were included for the study and were labeled from A1 to A29 in alphabetical order of the lead author's family name (or name of institution in cases of reports). The characteristics of each study, organized with the given study IDs (i.e., A1-A29), are presented in Table 1. A total of five studies were included as preprints during the literature search process. Four were later updated and included as publications in 2022 (A9, A11, A17, A23). One literature that was not updated was finally included as a preprint (A18). The number of studies released in 2021 were three times more than that of studies released in 2020 (76% and 24%, respectively). The literature was largely focused on the region of Africa (16 studies), followed by Asia (three studies) and both Africa and Asia (three studies). Seven studies were not confined to specific countries and/or broadly examined multi-territorial effects. The types of study design were the following: two qualitative studies, seven quantitative non-randomized studies, five quantitative descriptive studies, three mixed methods studies, and twelve reports.

**Table 1:** Characteristics of included studies

Study ID	Authors, year of publication	Study design	Country	Intervention type	Outcomes
A1	Afai et al. 2021	MM	Mozambique	RDT	Cases and deaths increased compared to the same period of previous year
A2	Aïkpon et al. 2020	QL	Benin Republic	ITNs distribution	Accurate and efficient implementation of the ITN mass distribution campaigns
A3	AMP, 2020	Report	26 countries	ITN campaigns, budget revisions	Successful distribution of ITNs in 2020 (74%) which saved approximately 160,000 children
A4	Buonsenso et al. 2020	QN	Sierra Leone	*Not described*	Cases decreased but not significant No change of deaths
A5	Buonsenso et al. 2021	QN	Sierra Leone	CHWs, ITNs distribution, Systematic diagnosis	No significant drop of cases and deaths in children
A6	Engoba et al. 2021	QN	DRC	Clinical interventions	Increased morbidity and mortality of malaria cases which is higher than those of COVID-19
A7	Feldman et al. 2021	QL	Cambodia	VMWs, MMWs, MPs	No decline of malaria service intervention coverage and utilization
A8	Gavi et al. 2021	QN	Zimbabwe	IRS, ITNs distribution	Increased cases and deaths coincided with COVID-19
A9	Hakizimana et al. 2022^a^	MM	Rwanda	LLINs, ANC and vaccination, IRS, HBM, CHWs, HCPs	Decreased tests in health facilities and increased in the community. Increased cases in the facility level, but no changed at the community level. No change of the trend in the number of deaths
A10	Hategeka et al. 2021	QN	DRC	*Not described*	Decreased cases and tests
A11	Heuschen et al. 2022^a^	QN	Ghana	Sufficient ACT, ITNs, health facility access	Decreased cases rebounded at the end of 2020
A12	Ilesanmi et al. 2021	QN	Nigeria	*Not described*	Decreased visits to healthcare facilities. Number of ITNs increased
A13	Mbunge et al. 2021	QN	Zimbabwe	IRS	Delayed IRS activities. Increased cases and deaths
A14	Namuganga et al. 2021	QN	Uganda	Case management with AL, treatment during ANC, LLINs distribution, RDT, IRS campaigns	Decreased RDT. No change of cases
A15	PMI, 2021	Report	Multiple	Multiple types	Disruptions in basic malaria services in health facilities and communities
A16	Seboka et al. 2021	QN	Africa 20 countries	ITN ownership, malaria testing	Decreased household ITN ownership rating, No significant impact on malaria testing utilization
A17	Suiyanka et al. 2022^a^	QN	Kenya	Mitigation, supply and distribution interruptions on the delivery of LLINs	LLIN campaign temporarily interrupted but caught up later
A18	Thapa et al. 2020—preprint	QN	Myanmar	Health service delivery system	Increased tests, decreased cases, timely distribution of LLINs
A19	The Common- wealth, 2021	Report	25 countries	Multiple types	Halted/delayed scheduled of LLIN mass campaigns, malaria commodities stock-out
A20	The Global Fund, 2020	Report	Multiple	Multiple types	Disruptions of malaria service delivery, delays in mosquito net distribution and indoor spraying programs
A21	The Global Fund, 2021a	Report	107 countries	Multiple types	Decreased tests and cases, mosquito nets campaigns initially delayed but rebounded
A22	The Global Fund, 2021b	Report	Africa 24, Asia 7 countries	Health service delivery system	Surveillance activities fallen in 2020, decreased cases in Africa and Asia, stock out of the antimalarial medicine in Africa
A23	Ward et al. 2022^a^	MM	Nigeria, Burkina Faso, Chad	Delivering SMC	Disrupted SMC delivery. Community distributors' increased workload for adhering to COVID-19 infection prevention and control measures
A24	WHO, 2020a	Report	105 countries	Health service delivery system	46% of the 68 countries reported that malaria diagnosis and treatment were disrupted
A25	WHO, 2020b	Report	Multiple	Multiple types	Disrupted ANC services, malaria diagnosis and treatment, reduction in all-cause outpatient attendance, decreased overall attendance at public health facilities, higher malaria cases
A26	WHO, 2021a	Report	Multiple	Diagnosis, treatment, prevention	Decreased tests, disrupted ITNs distribution, IRS campaigns, and SMC campaigns
A27	WHO, 2021b	Report	Multiple	Multiple types	Malaria prevention services (ITNs, IRS, and SMC distribution), reduction in outpatient attendances and malaria testing during the initial phase of the pandemic
A28	WHO, 2021c	Report	21 countries	Multiple types	Increased cases
A29	Wu et al. 2021	Report	China	Screening	No secondary cases caused by imported malaria

*QL* qualitative, *QN* quantitative, *MM* mixed methods, *AMP* the alliance for malaria prevention, *PMI* US President’s Malaria Initiative, *WHO* World Health Organization, *DRC* Democratic Republic of the Congo, *CHWs* community health workers, *AL* artemether-lumefantrine, *RDT* rapid diagnostic test, *ITN* insecticide-treated bed net, *ANC* antenatal care, *IRS* indoor residual spraying, *HBM* home based management of malaria, *SMC* seasonal malaria chemoprevention, *LLIN* long lasting insecticidal net, *MMWs* mobile malaria workers, *MPs* malaria posts, *VMWs* village malaria workers, *ACT* artemisinin-based combination therapy, *HCPs* health care provider

^a^It was preprinted document when this systematic review was initially conducted with the documents released in 2020–2021. Then some of the data has been updated later after it was published

Most of the included studies reported that the impact of the pandemic on malaria control was negative (22 out of 29 studies) in each study setting. Negative outcomes represent an increase in cases and deaths of malaria due to the pandemic compared to previous years (A1, A6, A8, A13, A28). Some studies report a decreased number of cases, diagnostic tests, or health facility visits temporarily following the start of the pandemic due to institution of lockdown measures (A10, A11, A12, A15, A22, A26, A27). Researchers confirmed negative outcomes to prevention and control measures for malaria by not only examining health metrics but also by examining the vector control programs, campaigns, and delayed basic services (A15, A16, A19, A20, A22, A23, A24, A25, A26, A27). Meanwhile, some studies reported how challenges were overcome over time. For example, challenges in distributing mosquito nets at the beginning of the pandemic were severe, but difficulty gradually decreased over time (A2, A17, A18, A21). Seven studies demonstrated no significant changes of malaria health outcomes during the pandemic, indicating no impact of COVID-19 on malaria (A3, A4, A5, A7, A9, A14, A29).

As previously mentioned a total of 27 barriers were identified and categorized into the four dimensions of access to healthcare (accessibility, affordability, availability, and acceptability) and further subdivided by the supply and demand sides (Table 2). Regardless of the subclassification into the supply and demand sides, the availability dimension in Table 2 encompasses almost half of all identified barriers (13 out of 27), followed by acceptability with seven barriers, geographical accessibility with four barriers, and affordability with three barriers. The supply side experienced more than double the barriers (a total of 19 barriers) compared to the demand side (a total of eight barriers). On the demand side, most notably indicating accessibility issues, over half of the studies indicated fear of contracting the coronavirus as a major barrier to malaria control.

**Table 2:** Barriers to malaria control during the COVID-19 pandemic

Dimensions	Barriers on supply side	Barriers on demand side
Geographic accessibility	Travel restrictions^a^: A3, A4, A5, A13, A20, A28 Lack of transportation^b^: A9, A19, A22	Travel restrictions^a^: A1, A3, A4, A8, A10, A11, A12, A13, A16, A19, A24, A28 Lack of transportation^b^: A9, A10, A25
Affordability	Increased costs for malaria control: A25, A27 Limited fiscal space/funding: A13, A25	Economic damage from COVID-19: A4, A9, A24
Availability	Procurement or supply chain issue: A6, A8, A9, A10, A11, A12, A13, A14, A15, A17, A19, A22, A24, A25, A27 Delayed or suspended ongoing malaria control intervention due to COVID-19 quarantine measures: A8, A9, A11, A13, A17, A20, A22, A24, A26, A28 The shift of resource or healthcare workforce from malaria to COVID-19: A2, A5, A6, A11, A16, A20, A25 Staff absences with sick, death, quarantine, or strikes related to COVID-19: A17, A18, A20, A22, A25 Insufficient personal protective equipment for health workers: A2, A9, A22, A25 Disrupted case management: A6, A8, A15 Clinical manifestations overlap between Malaria and COVID-19: A14, A22, A29 Postponement of recruitment or training of workforce: A13 Closure of health facilities^c^: A24 Poor program promotion, awareness, and marketing strategies: A13	Misled health advice disrupting timely management: A1, A11, A13, A25 Lack of information or knowledge on Malaria and/or COVID-19: A6, A9, A10 Closure of health facilities^c^: A12, A16
Acceptability	Fear of COVID-19 infection^d^: A9, A12, A18, A20, A23, A25 Lack of community’s engagement in malaria campaign: A13, A23 Lack of political will/support in malaria control: A13 Fear of violence targeting health workers (assumable due to COVID-19 stigmatization): A22 Difficulties in adhering to COVID-19 hygiene measures: A23	Fear of COVID-19 infection^d^: A1, A5, A7, A8, A9, A10, A11, A15, A16, A18, A21, A23, A25, A28 COVID-19 stigma: A10

^a^Travel restrictions: On the supply side, travel restrictions made health workers unable to provide malaria services. On the demand side, it disrupted access to malaria services

^b^Lack of transportation: On the supply side, lack of transportation made health workers absent from work. On the demand side, it limited access to uptake services

^c^Closure of health facilities: On the supply side, closure of health facilities affected normal operation of health service. On the demand side, it limited people to use health services

^d^Fear of COVID-19 infection: On the supply side, fear made health workers being passive in delivering malaria service. On the demand side, it made people less active in seeking health service

A total of 38 strategies were reported and, likewise, categorized into the four dimensions of access to healthcare (accessibility, affordability, availability, and acceptability) and further subdivided by the supply and demand sides (Table 3). The number of strategies for each dimension was similar to that of the number of barriers. Among all the strategies, 27 strategies were for the supply side while seven were for the demand side. Among the reported strategies, six strategies dealt with cross-cutting issues—applicable to all four dimensions. Below are detailed descriptions of the main findings by each of the four dimensions of the modified analytical framework employed in the study.

**Table 3:** Strategies for malaria control during COVID-19 pandemic

	Strategies on supply side	Strategies on demand side
Geographic accessibility	Non-contact monitoring and management methods for distribution of ITNs: A2, A15, A18, A19 Malaria diagnosis integrated with COVID-19 screening process^a^: A1, A12 Technical innovation in malaria interventions and approaches^a^: A19, A25 Allowing healthcare workers’ travel during lockdown: A14	Mobilizing community health workers for malaria intervention^a^: A1, A7, A9, A20, A21, A22 Door-to-door distribution of ITNs^a^: A2, A15, A19, A20, A22 Enhancing community-based malaria control: A5, A9, A25
Affordability	Expanding investment in malaria intervention: A16 Continuous financial support from external partners: A2, A3, A18, A22	
Availability	Maintaining essential healthcare services during COVID-19: A2, A6, A7, A8, A10, A14, A16, A24, A26 Strengthening data and surveillance system: A14, A21, A22, A25, A29 Using PPE when conducting malaria intervention: A2, A7, A18, A20, A22 Integrating malaria interventions with existing regular healthcare: A5, A23 Malaria diagnosis integrated with COVID-19 screening process^a^: A1, A12 Technical innovation in malaria interventions and approaches^a^: A19, A25 Coordination of medicine procurement and logistic system: A15, A22 A comprehensive approach that aims to provide COVID-19 care: A4 Facilities as well as social services and essential resources: A4 Real-time monitoring of changes and rapid response to circumstances: A7 Modification of malaria interventions adapted to COVID-19 measures: A11 Establishing a commodity tracking system: A19 Enhancing health workforce and malaria experts: A25 Conducting a modelling analysis to grasp the impact of COVID-19 on the burden of malaria: A27	Mobilizing community health workers for malaria intervention^a^: A1, A7, A9, A20, A21, A22 Enhancing community-based health system and building systematic resilience: A5, A9, A17, A20, A21, A22, A25 Door-to-door distribution of ITNs^a^: A2, A15, A19, A20, A22
Acceptability	Establishing guidelines for malaria intervention during COVID-19: A3, A7, A9, A15, A18, A23, A27 Applying Trust, Relevance, and Connection management strategy for malaria control: A7 Continual communication and monitoring: A3	Disseminating correct information about COVID-19 and/or malaria: A5, A7, A9, A23 Delivering information on malaria intervention using mobile technology and/or ICT: A2, A13 Health education and promotion for community people using advertisement, radio and social media: A15, A23
Cross-cutting for all 4 dimensions	Continuous technical support from external partners: A2, A3, A18, A22, A28 The government’s strong political commitment and support: A2, A5, A28 Improved ownership, leadership and management at all governmental levels and sectors: A12, A19, A25 Strategic partnerships across sectors and effective coordination: A2, A7 Cross-border collaboration: A19 Rapid problem-solving: A2	

*RDT* rapid diagnostic test, *ICT* information and communications technology, *ITN* insecticide-treated net, *PPE* personal protective equipment, *RT-PCR* reverse transcription polymerase chain reaction

^a^Classified as corresponding to both geographic accessibility and availability dimensions

### Geographic accessibility

The first dimension, geographical accessibility, was a significant factor. Inability to physically move from one place to another greatly affected malaria control. Travel restriction served as a critical barrier for both supply and demand sides (Table 2). In order to stop the spread of the coronavirus, many governments-imposed lockdown measures, which led to health services disruptions. A total of 14 studies reported travel restrictions as part of lockdown measures as a barrier of malaria service provision or uptake (A1, A3, A4, A5, A8, A10, A11, A12, A13, A16, A19, A20, A24, A28).

Lack of transportation was another challenge to malaria control for both supply and demand sides (A9, A10, A19, A22, A25). For example, in Rwanda, measures to curb the impact of the COVID-19 pandemic limited the availability of transportation, resulting in delays in delivery and utilization of malaria service (A9).

For improvements in geographic accessibility, strategies for supply side encompassed technical innovation in malaria interventions and approaches (A19, A25), allowing healthcare workers to travel during lockdown (A14), non-contact monitoring and management methods for distribution of insecticide-treated nets (ITNs) (A2, A15, A18, A19), and malaria diagnosis linked with COVID-19 screening (A1, A12). Strategies for demand side included mobilizing community health workers for malaria intervention (A1, A7, A9, A20, A21, A22), door-to-door distribution of ITNs (A2, A15, A19, A20, A22), and enhancements in community-based malaria control (A5, A9, A25).

### Affordability

COVID-19 affected the affordability dimension as well. Economic damage was the main barrier for people on the demand side as opportunities to earn money were taken away due to COVID-19 (A4, A24). Lockdown measures to curb coronavirus affected people’s socioeconomic statuses, creating financial barriers that discouraged them to seek health services (A9, A24). Financial barriers prevented patients from getting timely treatment. For example, transportation fees and visit fees for malaria services were a substantial burden to people in rural areas of Sierra Leone (A4).

Additionally, increased costs for malaria control made it difficult for healthcare professionals to provide care (A25, A27). Challenges in the global supply chain contributed to the increased price of raw materials for malaria commodities and freight (A25, A27).

Also, during the pandemic, funding for malaria control became inconsistent, and the healthcare workforce and resources initially allocated to malaria prevention efforts were re-allocated to COVID-19 (A13, A25). In Zimbabwe, malaria cases increased exponentially in 2020. Experts concluded that the increase was primarily due to the lack and inconsistency of funding allocated towards malaria control (A13). The strategies to improve affordability dimension are crucial to ensure more long-term approaches for pandemic preparedness. For both supply and demand sides, expanding investments in malaria intervention (A16) and continuous financial support from external partners (A2, A3, A18, A22) are suggested as strategies for the affordability dimension.

### Availability

The COVID-19 pandemic most heavily affected availability. For this, many factors were considered. From the supply side, essential health services and malaria control interventions were disrupted (A8, A9, A11, A13, A17, A20, A22, A24, A26, A28). The World Health Organization (WHO)’s survey assessing the impact of the pandemic on essential health services in 2020 showed that almost half of the responses reported disruptions in malaria diagnosis and treatment during the pandemic (A24). A follow-up survey conducted in 2021 detailed the features of the disruptions. Disruptions were identified in providing diagnosis and treatment, distributing ITNs, indoor residual spraying (IRS), and seasonal malaria chemoprevention (SMC), making up between 30 and 40% of disruptions to the implementation of those services in malaria endemic countries (A26). Especially the widely implemented vector control activities, such as ITN campaign or long-lasting insecticidal nets (LLINs) and IRS distribution, were hindered (A9, A11, A13, A17, A20, A26, A28). Additionally, the COVID-19 lockdowns and travel restrictions are noted as reason of delays in many different countries, including but not limited to IRS and LLINs distribution in Rwanda (A9), ITN distribution during delivery of antenatal care (ANC) in Ghana (A11), IRS activities in Zimbabwe (A13), and canceled LLINs distribution campaign in Western Kenya (A17). The aforementioned cases indicate that the delay in malaria control is closely interlinked with the onset of the pandemic. Limited access to and delivery of antenatal care interrupted the routine provision of IPTp (Intermittent preventive treatment of pregnancy) to the women in the Northern region of Ghana, resulting in the decline of malaria patients in March and April 2020 (A11). The trend bounced back after lifting movement restrictions. The delay in hiring and training the workforce (A13) and closure of health facilities (A12, A16, A24) are other barriers that negatively affected malaria control efforts.

Specifically, supply chain issues were reported. Procuring commodities for malaria control and equipment such as mosquito nets, sprays, medicines were difficult (A6, A8, A9, A10, A11, A12, A13, A14, A15, A17, A19, A22, A24, A25, A27). The pandemic challenges the logistics of delivering malaria products between and within countries, resulting in delayed procurement of items for vector control such as ITN, LLIN, and IRS (A25). For example, in Uganda and the Democratic Republic of the Congo (DRC), the decrease in the number of patients who tested for malaria in 2020 is attributed to the disrupted global supply chain of malaria commodities that resulted in lack of local stock of malaria test kits (A10, A14). While Nigeria reported that limited procurement of malaria tests to healthcare providers was a barrier against visiting health facilities (A12), the Global Fund Results Report highlights the increased demand for COVID-19 tests and vector control products in the market disrupted the supply chain of malaria products (A22).

Amidst the pandemic, both resources and the healthcare workforce initially dedicated to malaria control was re-allocated to COVID-19 prevention measures (A2, A5, A6, A11, A16, A20, A25). Allocation of resources for COVID-19 control were prioritized, resulting in neglect in malaria control and its preventive measures such as essential tools (A6, A11, A16). Additionally, the delivery of health service was weakened due to limited personnel available. Many studies report unavailability of healthcare workers because many had fallen ill, died, were subject to quarantine, and/or engaged in COVID-19 strikes (A17, A18, A20, A22, A25).

Insufficient resources for healthcare workers became a barrier as well. The most notable barrier was a lack of personal protective equipment (PPE). PPEs such as masks were unavailable and insufficient and their delivery was delayed to healthcare workers at the frontline of the responses (A2, A9, A22, A25). The shortage of PPE sometimes resulted in healthcare professionals refusing to physically take care of patients (A9).

Among the general population, it was reported that the lack of information and knowledge regarding the two diseases, malaria and COVID-19, was a barrier to utilizing health services. Because patients were unaware of the serious symptoms and signs of infection, consultations were delayed (A6). One study noted how because of the shift in focus to the COVID-19 pandemic, people were less likely to get the information they needed on malaria (A9). In Gombe, DRC, for example, even though hospitals and health clinics remained open and functional, a lack of clear information on the lockdown transportation and activity policies hindered people from using health services (A10). Furthermore, lack of trustworthy health messages was a barrier when looking for health advisement (A1, A11, A13, A25). At the beginning of the COVID-19 pandemic, the media propagated “stay home” messages to help curb the spread of the coronavirus. This, however, worsened health conditions of people with malaria (A1, A11). COVID-19 and malaria share many similar symptoms like having a fever. And because the large bulk of the general population was unable to distinguish between the two, many were told to stay home (A11, A13, A25), negatively affecting malaria control and prevention efforts.

On the supply side, case management was heavily disrupted by the pandemic. Lack of management in medical records to keep track of patients and changes in medical practices and case management further negatively affected the already fragile health systems (A6, A8, A15). Also, as mentioned before, similar onset of symptoms between malaria and COVID-19 made it harder for the healthcare providers to distinguish between the two and make a correct prompt diagnosis (A14, A23, A29). In Uganda, the overlapping symptoms such as fever led to an increase of suspected cases and supply demand for malaria, resulting in overdiagnosis and overtreatment (A14). The literature indicates that the impact of COVID-19 on preventative measures. It is widely known that public campaigns to raise awareness of malaria play an important role. However, during the pandemic, campaigns, program promotion, awareness and marketing strategies were affected (A13).

As shown in Table 3, there are 17 strategies for the availability dimension that were either actually implemented or suggested from the selected documents. Notably, a majority of strategies for the availability dimension are recommended for the supply side and they include the following: maintaining essential healthcare services during a pandemic situation like the COVID-19 pandemic (A2, A6, A7, A8, A10, A14, A16, A24, A26), strengthening data and surveillance systems (A14, A21, A22, A25, A29), using PPE when conducting malaria intervention (A2, A7, A18, A20, A22), integrating malaria interventions with existing regular healthcare activities (A5, A23), implementing technical innovation in malaria interventions and approaches (A19, A25), and enhancing health workforce and malaria experts (A25). Further, some studies (A1, A5, A12, A23) suggested combining healthcare services (e.g., COVID-19 prevention measures) and malaria control. Another study (A4) recommended providing not only health services but also social services.

The three strategies recommended for the demand side are: mobilizing community health workers or malaria intervention (A1, A7, A9, A20, A21, A22), enhancing community-based health systems and building systematic resilience (A5, A9, A17, A20, A21, A22, A25), and conducting door-to-door distribution of ITNs (A2, A15, A19, A20, A22).

### Acceptability

Acceptability refers to how people react to COVID-19 and malaria control measures. Fear of contracting COVID-19 was seen on both sides. While healthcare providers feared getting infected at the workplace, the general population feared getting infected upon their visit to healthcare facilities (A1, A5, A7, A8, A9, A10, A11, A12, A15, A16, A18, A20, A21, A23, A25, A28). In Sierra Leone and Myanmar, people were afraid of contracting the virus at health centers (A5, A18, A21). In Ghana, pregnant women were hesitant to utilize health facilities because they feared exposure to COVID-19 (A11). In Kinshasa, the use of treatment centers declined, and many studies attribute such a reduction to the fear of contracting COVID-19. The literature is prolific in documenting the stigmatization of health centers (A10). Consequently, many healthcare providers feared getting attacked due to COVID-19 stigmatization (A22).

Malaria campaigns are important when it comes to malaria prevention measures, but the community's engagement was low (A13, A23). In Nigeria, Burkina Faso, Chad and Zimbabwe, the community’s engagement in malaria campaigns was restricted due to COVID-19. For example, Ward et al. (2021), reported difficulty implementing door-to-door delivery of SMC (A23). Furthermore, lack of political will was one of the challenges of malaria elimination strategies (A13).

Adhering to COVID-19 hygiene measures seemed to be another challenge because community health workers hardly followed hygiene rules like thirty-second hand washing, safe distancing and thermal screening. According to a study conducted in three African countries—Nigeria, Burkina Faso and Chad—community distributors rarely followed the recommended thirty-second hand washing rule in Nigeria and Burkina Faso. Safe distancing and thermal screening were not explicitly practiced as recommended for infection prevention (A23).

Strategies for resolving the acceptability problems focused on increasing awareness and transmission of appropriate communication. For the supply side, the following strategies were identified: establishing guidelines for malaria intervention during COVID-19 (A3, A7, A9, A15, A18, A23, A27), applying trust, relevance, and connection management strategies for malaria control (A7) and continuing communication and monitoring (A3). For the demand side, disseminating correct information about COVID-19 and malaria (A5, A7, A9, A23), delivering information on malaria intervention using mobile technology and/or ICT (A2, A13) and utilizing social media for health education and promotion for community (A15, A23) are appropriate strategies. As acceptability issues are accounted for both supply and demand sides, the strategies, likewise, are evenly reported.

Additionally, there are six cross-cutting strategies reported throughout the studies that are worth noting for addressing barriers in all the dimensions (Table 3). All of them are applicable for both supply and demand sides: (1) continuous technical support from external partners and international cooperation (A2, A3, A18, A22, A28), (2) strong political commitment and interest from the government to support building resilient society from the pandemic (A2, A5, A28), (3) improved ownership, leadership and management at all governmental levels and sectors for active engagement (A12, A19, A25), (4) strategic partnerships across various sectors and effective coordination (A2, A7), (5) cross-border collaboration (A19), and (6) rapid problem-solving culture for efficient decision-making process (A2).

**Fig. 2:**
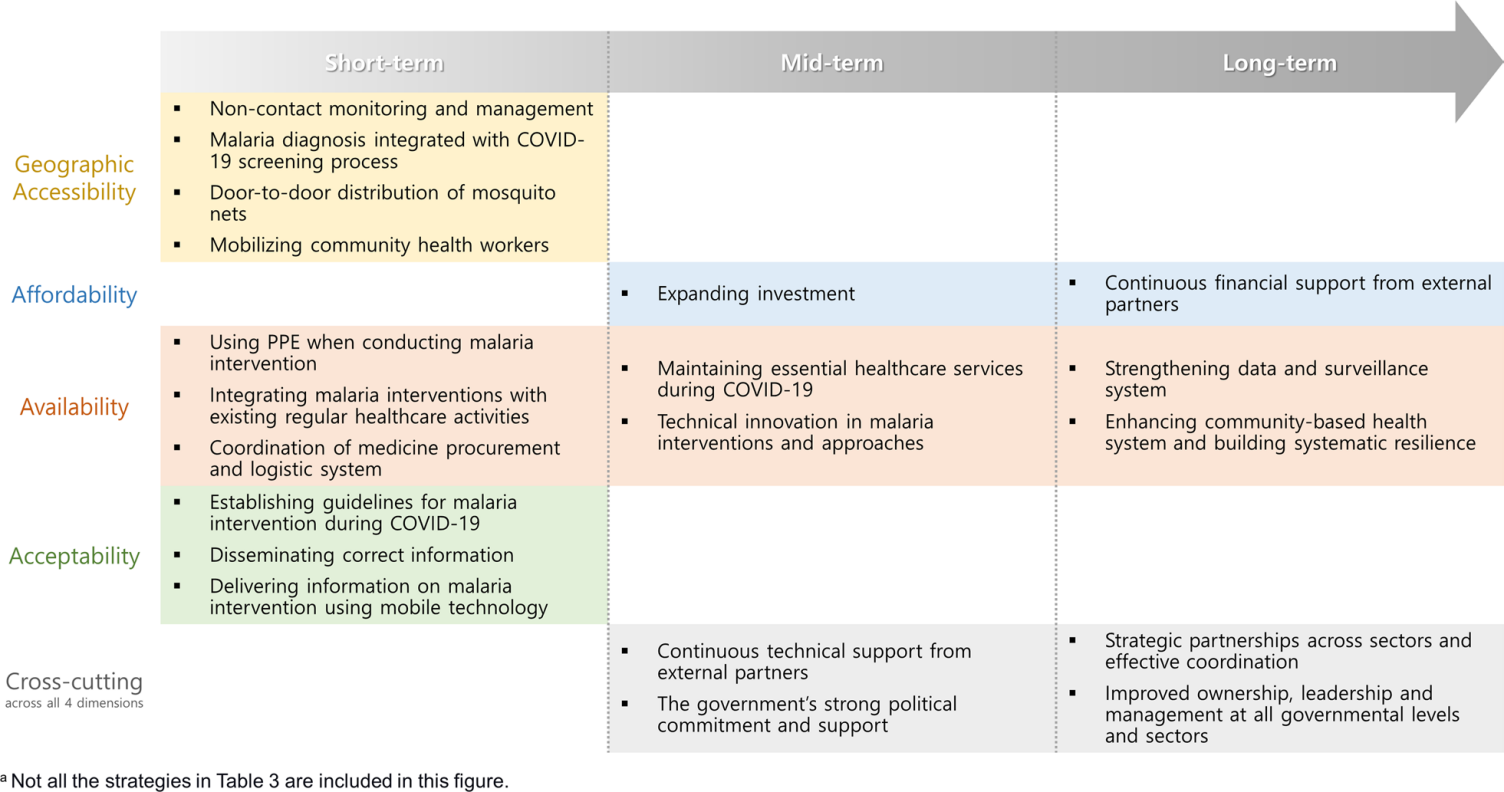
Strategies classified in terms of the time frame

Moreover, the strategies are divided into three phases (i.e., short-term, mid-term, and long-term) based on the time it takes for the goals to be achieved (Fig. 2). Among the four dimensions, geographic availability and acceptability are more geared towards short-term, focusing on instant response to COVID-19. Availability dimension requires both long-term and short-term strategies simultaneously. Short-term strategies alone could help overcome current barriers; however, they are ultimately helpful for health system strengthening. As for the affordability dimension, strategies appear to be lacking and inconclusive.

In order for the strategies to work effectively, financial resources and long-term planning are necessary. As there are many barriers reported for the availability dimension, there are many suggested strategies as well compared to other dimensions. Several dimensions in blue are helpful for both geographic accessibility and availability and they can be interchangeably used.

## Discussion

Barriers to and strategies for the provision and uptake of malaria services during the pandemic in LMICs were identified and categorized into the four dimensions of access to healthcare: accessibility, affordability, availability, and acceptability. During a pandemic, barriers and strategies cannot be addressed with a silo approach. Thus, this study contributes by identifying barriers and strategies through a systematic literature review by using a single framework, helping to further facilitate the provision of an integrated approach addressing the different dimensions as well as the timeframe of short and long-term strategies.

The categorization of barriers facilitates the understanding of the contexts in which these barriers develop during the pandemic. Firstly, COVID-19 lockdown measures have directly limited geographic accessibility of people and resources. Travel restrictions or lack of transportation deterred people in both supply and demand sides from reaching service delivery points at both country and international levels. Increased cost of the shipment and raw materials and disproportionate economic impact of unemployment in vulnerable populations of Africa further complicated responses. Provision of proper funds was disrupted as well. Notwithstanding, community-level approaches such as mobilizing community health workers and employing non-contact and integrated methods could mitigate the geographic barriers. Secondly, weak health systems in LMICs were insufficient to respond to the ongoing rise of additional demands of the pandemic. Reduction in health workers and resources, poor supply chain and complications in logistics resulted in delayed or suspended malaria control activities. Malaria control, alongside COVID-19 responses, would be possible with strengthening of health care system to facilitate essential services as well as surveillance. Lastly, psychosocial factors changed people’s behaviors, affecting malaria control measures. The “fear” of contracting the coronavirus increased hesitation in visiting health facilities, fear of violence targeting health workers (assumable due to COVID-19 stigmatization) and decreased community support. These barriers can be ameliorated through clear risk communication strategies.

Potential strategies to overcome the above-mentioned barriers were gathered and classified into three phases (i.e., short-, middle- and long-term) based on the time needed for implementation. Most strategies identified were short- and middle-term strategies. However, much of the analysed literature highlighted the necessity of health systems strengthening in LMICs as a long-term strategy to avoid disruptions in the provision of essential healthcare services including malaria services. This means, among the observed barriers, structural problems such as facility closure, insufficient personnel, and financial barriers have appeared repeatedly—as was the case for the Ebola outbreak [18]. Therefore, there is an urgent need of constructing strategies for health system strengthening in the long term to make LMICs more prepared and resilient [19, 20]. Moreover, implementation of health systems strengthening should be balanced between building blocks of health systems to overcome a silo approach [21]. Not surprisingly, financing, information, and leadership and governance, which are conventionally neglected in strengthening health systems [22], are typically suggested long-term strategies among others to overcome barriers to malaria intervention during the pandemic. Hence, it may be the time to reiterate systems thinking in health systems strengthening amid fragmented and short-sighted approaches [23].

In addition to strengthening the national health system, a well-integrated approach should be considered to deliver resilient health services during the pandemic [24]. For example, malaria messages and diagnosis are suggested to be integrated in COVID-19 responses to release pressure on health systems amid the pandemic. This study identified several cross-cutting strategies at systems-level integration, such as political leaderships, collaboration with external partners, and whole-of-government approach, that will help LMICs to integrate healthcare services, be more resilient, and overcome barriers to access to malaria services in all dimensions. Still, these identified strategies are limited to high level politics. This may result in neglect in voices from the “bottom” and on-the-ground [17]. Therefore, integrated health service delivery should engage the whole community to pursue people-centeredness in health systems [25]. Moreover, country-specific solutions should be considered [26]. This suggestion is also aligned with recent strategies at the global level. The recently revised Global Technical Strategy (GTS) for malaria 2016–2030 and global actors reiterated national ownership, leadership, intervention suitable for the local environment, and equity in an access to services as basic principles of malaria intervention [10].

The impact of the novel virus like COVID-19 presents an opportunity for integrating approaches through strengthening national government’s capacity and encouraging community engagement. Therefore, it is imperative that lessons learned from past infectious diseases are internalized and applied to better prepare against unknown future pandemics [18]. While it is still worthy to adopt and implement short-term strategies and technical adjustments in malaria intervention to better respond to the instant increase in the number of cases and deaths of malaria, “the role of health structures, systems and staff” should be prioritized as a core aspect for malaria control in long-term solution as guided by WHO [27].

This systematic review has the following limitations. First, this review takes into consideration a large bulk of unpublished literature (i.e., preprints and reports from renowned institutions) to encompass up-to-date information on malaria responses during the pandemic. Therefore, the findings of this research should be interpreted carefully as preprint literature can change. Further, some of the reports were concerned with low accuracy in the quality appraisal assessment. Second, the study is rather limited in drawing a causal relationship between identified barriers and disrupted malaria services because of the presence of indirect factors affecting malaria control (e.g., heavy rain, seasonal variation, or population movement). Therefore, one should avoid extrapolating results to a specific country without considering differences in healthcare capacity, cultural context, and the pandemic consequences. Further research might be needed to qualitatively identify deep-rooted causes of disrupting malaria control and prevention efforts. Third, identified strategies are not exhaustive. Published strategies may also be biased as small number of powerful actors in malaria control at the global level are responsible for suggesting them. This may be because capacity and efforts to identify evidence-based strategies to tackle the malaria burden are limited during the health crisis. The world should support generating empirical studies to make evidence-driven policy recommendations [28]. Lastly, since this study conducted an analysis focused on the barriers and strategies during the pandemic, the impact on health outcomes related to malaria disease have not been thoroughly analysed in a quantitative and qualitative spectrum. Thus, further research should be conducted for a more complete understanding of the topic.

## Conclusion

Identifying barriers that hinder malaria control in LMICs during the COVID-19 pandemic and strategies to reduce the barriers provides useful insights to help decision-making process in current and future malaria control. This study highlights cross-cutting strategies that embrace and integrate all dimensions of access to healthcare. Health system strengthening and resilience strategies appropriate for various country-specific contexts is fundamental to effectively deal with the ongoing pandemic. The short-term strategies should include the continuum of public health care while the mid- to long-term approaches require political will and financial support. The COVID-19 pandemic is an unprecedented global health threat, but, at the same time, it provides a unique opportunity to comprehensively integrate different approaches, including strengthening healthcare capabilities of governments and encouraging active community engagement. It is crucial to be prepared for future pandemics by reflecting on the lessons learned from the past and present infectious disease crises.

## Selected studies

1. Afai G, Banze, AR, Candrinho B, Baltazar CS, Rossetto EV. Challenges for malaria surveillance during the COVID-19 emergency response in Mampula, Mozambique, January-May 2020. Pan African Medical Journal. 2021;38:254. https://doi.org/10.11604/pamj.2021.38.254.27481
2. Aïkpon R, Affoukou C, Hounpkatin B, Eclou D-D, Cyaka Y, Egwu E, et al. Digitalized mass distribution campaign of insecticide-treated nets (ITNs) in the particular context of Covid-19 pandemic in Benin: challenges and lessons learned. Malar J. 2020;19(1):431. https://doi.org/10.1186/s12936-020-03508-x
3. The Alliance for Malaria Prevention. Annual Report 2020. 2020. Available from: https://allianceformalariaprevention.com/wp-content/uploads/2021/03/FINAL-AMP-Annual-Report-2020.pdf
4. Buonsenso D, Cinicola B, Kallon MN, Iodice F. Child healthcare and immunizations in Sub-Saharan Africa during the COVID-19 pandemic. Front Pediatr. 2020;8:517. https://doi.org/10.3389/fped.2020.00517
5. Buonsenso D, Iodice F, Cinicola B, Raffaelli F, Sowa S, Ricciardi W. Management of malaria in children younger than 5 years old during coronavirus disease 2019 pandemic in Sierra Leone: a lesson learned? Front Pediatr. 2021;8:587,638. https://doi.org/10.3389/fped.2020.587638
6. Engoba M, Gnakingue ANO, Nianga BB, Goma CEG, Batchi-Bouyou AL, Okoko AR, et al. Impact of the Covid-19 pandemic on severe childhood malaria at the university hospital of Brazzaville. Open Journal of Pediatrics. 2021;11(2):301–12. https://doi.org/10.4236/ojped.2021.112028
7. Feldman M, Vernaeve L, Tibenderana J, Braack L, Debackere M, Thu HK, et al. Navigating the COVID-19 crisis to sustain community-based malaria interventions in Cambodia. Global Health: Science and Practice. 2021;9(2):344–54. https://doi.org/10.9745/GHSP-D-20-00528
8. Gavi S, Tapera O, Mberikunashe J, Kanyangarara M. Malaria incidence and mortality in Zimbabwe during the COVID-19 pandemic: analysis of routine surveillance data. Malaria J. 2021;20(1):233. https://doi.org/10.1186/s12936-021-03770-7
9. Hakizimana D, Ntizimira C, Mbituyumuremyi A, Hakizimana E, Mahmoud H, Birindabagabo P, et al. The impact of Covid-19 on malaria services in three high endemic districts in Rwanda: a mixed-method study. Malar J. 2022;21(48). https://doi.org/10.1186/s12936-022-04071-3
10. Hategeka C, Carter SE, Chenge FM, Katanga EN, Lurton G, Mayaka SM-N, et al. Impact of the COVID-19 pandemic and response on the utilisation of health services in public facilities during the first wave in Kinshasa, the Democratic Republic of the Congo. BMJ Glob Health. 2021;6(7):e005955. https://doi.org/10.1136/bmjgh-2021-005955
11. Heuschen A-K, Abdul-Mumin A, Adokiya MN, Lu Guangyu, Jahn A, Razum O, et al. Impact of the COVID-19 pandemic on malaria cases in health facilities in northern Ghana: a retrospective analysis of routine surveillance data. Malar J. 2022;21(1):48. https://doi.org/10.1186/s12936-022-04154-1
12. Ilesanmi OS, Afolabi AA, Iyiola OP. Effect of the COVID-19 pandemic on malaria intervention coverage in Nigeria: analysis of the premise malaria COVID-19 health services disruption survey 2020. Popul Med. 2021;3(September):24. https://doi.org/10.18332/popmed/141979
13. Mbunge E, Millham R, Sibiya N, Takavarasha S Jr. Is malaria elimination a distant dream? Reconsidering malaria elimination strategies in Zimbabwe. Public Health Pract. 2021;2:100,168. https://doi.org/10.1016/j.puhip.2021.100168.
14. Namuganga JF, Briggs J, Roh ME, Okiring J, Kisambira Y, Sserwanga A, et al. Impact of COVID-19 on routine malaria indicators in rural Uganda: an interrupted time series analysis. Malar J. 2021;20(1):475. https://doi.org/10.1186/s12936-021-04018-0
15. US President’s Malaria Initiative. 15 years of fighting malaria and saving lives. 2021. Available from: https://d1u4sg1s9ptc4z.cloudfront.net/uploads/2021/07/PMI-15th-Annual-Report-1.pdf
16. Seboka BT, Hailegebreal S, Kabthymer RH, Ali H, Yehualashet DE, Demeke AD, et al. Impact of the COVID-19 pandemic on malaria prevention in Africa: evidence from COVID-19 health services disruption survey. J Trop Dis. 2021;9(6):287. Available from: https://www.walshmedicalmedia.com/open-access/impact-of-the-covid19-pandemic-on-malaria-prevention-in-africa-evidence-from-covid19-health-services-disruption-survey-82309.html
17. Suiyanka L, Alegana VA, Snow RW. Insecticide-treated net distribution in western Kenya: impacts related to COVID-19 and health worker strikes. Int Health. 2022;14(5):537–539. https://doi.org/10.1093/inthealth/ihab051
18. Thapa B, Thi A,Than WP, Win KM, Khine SK. Myanmar continues to curb malaria amid coronavirus disease-2019 crisis. Res Sq. 2020. https://doi.org/10.21203/rs.3.rs-101547/v1
19. The Commonwealth. The Commonwealth Malaria Report 2021. 2021. Available from: https://malarianomore.org.uk/sites/default/files/Commonwealth%20Malaria%20Report%202021.pdf
20. The Global Fund. Mitigating the impact of COVID-19 on countries affected by HIV, tuberculosis and malaria. Global Health Campus: Geneva. 2020. Available from: https://www.theglobalfund.org/media/9819/covid19_mitigatingimpact_report_en.pdf
21. The Global Fund. Results Report 2021. 2021. Available from: https://www.theglobalfund.org/media/11304/corporate_2021resultsreport_report_en.pdf
22. The Global Fund. The impact of covid-19 on HIV, TB and malaria services and systems for health: a snapshot from 502 health facilities across Africa and Asia. Global Fund Geneva, Switzerland. 2021. Available from: https://www.theglobalfund.org/media/10776/covid-19_2020-disruption-impact_report_en.pdf
23. Ward C, Phillips A, Oresanya O, Olisenekwu G, Arogunade E, Moukénet A, et al. Delivery of seasonal malaria chemoprevention with enhanced infection prevention and control measures during the COVID-19 pandemic in Nigeria, Burkina Faso and Chad: a cross-sectional study. Malar J. 2022;21(1):103. https://doi.org/10.1186/s12936-022-04091-z
24. WHO. Pulse survey on continuity of essential health services during the COVID-19 pandemic: interim report, 27 August 2020. Geneva: World Health Organization; 2020. Available from: https://www.who.int/publications/i/item/WHO-2019-nCoV-EHS_continuity-survey-2020.1
25. WHO. World malaria report 2020: 20 years of global progress and challenges. Geneva: World Health Organization; 2020. Available from: https://www.who.int/publications/i/item/9789240015791
26. WHO. Second round of the national pulse survey on continuity of essential health services during the COVID-19 pandemic: January-March 2021: interim report, 22 April 2021. Geneva: World Health Organization; 2021. Available from: https://apps.who.int/iris/handle/10665/340937
27. WHO. World malaria report 2021. Geneva: World Health Organization; 2021. Available from: https://www.who.int/publications/i/item/9789240040496
28. WHO. Zeroing in on malaria elimination: Final report of the E-2020 initiative. Geneva: World Health Organization; 2021. Available from: https://www.who.int/publications/i/item/9789240024359
29. Wu D, Deng Z, Lin R, Mao Q, Lu W, Ruan C, et al. Malaria surveillance of entry people during the COVID-19 epidemic—Guangdong province, China, October 2020-May 2021. China CDC Wkly. 2021;3(38):799–802. https://doi.org/10.46234/ccdcw2021.180

## Supplementary Information

**Additional file 1: **Sample of search terms (PubMed).

**Additional file 2: **Quality appraisal results.

**Additional file 3: **PRISMA 2020 Checklist.

**Additional file 4: **PRISMA 2020 for Abstracts Checklist.

## Data Availability

All data generated or analysed during this study are included in this published article and its additional file.

## Abbreviations

AACODS: Authority, accuracy, coverage, objectivity, date and significance
ACT: Artemisinin-based combination therapy
AL: Artemether-lumefantrine
AMP: The alliance for malaria prevention
ANC: Antenatal care
CHWs: Community health workers
CINAHL: Cumulated index to nursing and allied health literature
COVID-19: Coronavirus disease 2019
DRC: Democratic Republic of the Congo
EMBASE: Excerpta Medica data BASE
EVD: Ebola virus disease
HBM: Home based management of malaria
HCPs: Health care providers
ICT: Information and communications technology
IPTp: Intermittent preventive treatment of malaria during pregnancy
IRS: Indoor residual spraying
ITN: Insecticide-treated net
LLIN: Long-lasting insecticidal net
LMICs: Low- and middle- income countries
MMAT: Mixed methods quality appraisal tool
MMWs: Mobile malaria workers
MPs: Mobile malaria posts
PMI: US President’s Malaria Initiative
PPE: Personal protective equipment
RT-PCR: Reverse transcription polymerase chain reaction
VMWs: Village malaria workers
WHO: World Health Organization
