# Supplementary material for: Barriers against and strategies for malaria control during the COVID-19 pandemic in low- and middle-income countries: a systematic review
Source: Malar J. 2023 Feb 3;22:41. doi: 10.1186/s12936-023-04452-2 (PMC9896667; doi:10.1186/s12936-023-04452-2)
Supplement: Supplementary file 1 — Additional file 1: Sample of search terms (PubMed). [file 12936_2023_4452_MOESM1_ESM.docx]

**Additional file 1. Sample of search terms (PubMed)**

| Set | Key words |
| --- | --- |
| #1 | "Deprived Countries"[All Fields] OR "Deprived Population"[All Fields] OR "Deprived Populations"[All Fields] OR "Developing Countries"[All Fields] OR "Developing Country"[All Fields] OR "Developing Economies"[All Fields] OR "Developing Economy"[All Fields] OR "Developing Nation"[All Fields] OR "Developing Nations"[All Fields] OR "Developing Population"[All Fields] OR "Developing Populations"[All Fields] OR "Developing World"[All Fields] OR "LAMI Countries"[All Fields] OR "LAMI Country"[All Fields] OR "less developed countries"[All Fields] OR "less developed country"[All Fields] OR "Less Developed Economies"[All Fields] OR "less developed nation"[All Fields] OR "less developed nations"[All Fields] OR "Less Developed World"[All Fields] OR "Lesser Developed Countries"[All Fields] OR "Lesser Developed Nations"[All Fields] OR "LMIC"[All Fields] OR "LMICS"[All Fields] OR "Low GDP"[All Fields] OR "Low GNP"[All Fields] OR "Low Gross Domestic"[All Fields] OR "Low Gross National"[All Fields] OR "low income countries"[All Fields] OR "Low Income Country"[All Fields] OR "Low Income Economies"[All Fields] OR "Low Income Economy"[All Fields] OR "Low Income Nations"[All Fields] OR "Low Income Population"[All Fields] OR "Low Income Populations"[All Fields] OR "Lower GDP"[All Fields] OR "lower gross domestic"[All Fields] OR "Lower Income Countries"[All Fields] OR "Lower Income Country"[All Fields] OR "Lower Income Nations"[All Fields] OR "Lower Income Population"[All Fields] OR "Lower Income Populations"[All Fields] OR "middle income countries"[All Fields] OR "Middle Income Country"[All Fields] OR "Middle Income Economies"[All Fields] OR "Middle Income Nation"[All Fields] OR "Middle Income Nations"[All Fields] OR "Middle Income Population"[All Fields] OR "Middle Income Populations"[All Fields] OR "Poor Countries"[All Fields] OR "Poor Country"[All Fields] OR "Poor Economies"[All Fields] OR "Poor Economy"[All Fields] OR "Poor Nation"[All Fields] OR "Poor Nations"[All Fields] OR "Poor Population"[All Fields] OR "Poor Populations"[All Fields] OR "poor world"[All Fields] OR "Poorer Countries"[All Fields] OR "Poorer Economies"[All Fields] OR "Poorer Economy"[All Fields] OR "Poorer Nations"[All Fields] OR "Poorer Population"[All Fields] OR "Poorer Populations"[All Fields] OR "Third World"[All Fields] OR "Transitional Countries"[All Fields] OR "Transitional Country"[All Fields] OR "Transitional Economies"[All Fields] OR "Transitional Economy"[All Fields] OR "under developed countries"[All Fields] OR "under developed country"[All Fields] OR "under developed nations"[All Fields] OR "Under Developed World"[All Fields] OR "Under Served Population"[All Fields] OR "Under Served Populations"[All Fields] OR "Underdeveloped Countries"[All Fields] OR "Underdeveloped Country"[All Fields] OR "underdeveloped economies"[All Fields] OR "underdeveloped nations"[All Fields] OR "underdeveloped population"[All Fields] OR "Underdeveloped World"[All Fields] OR "Underserved Countries"[All Fields] OR "Underserved Nations"[All Fields] OR "Underserved Population"[All Fields] OR "Underserved Populations"[All Fields] OR "low-income setting"[All Fields] OR "low-income settings"[All Fields] OR "low income countries"[All Fields] OR "middle-income setting"[All Fields] OR "middle-income settings"[All Fields] OR "middle income countries"[All Fields] OR "low-income setting"[All Fields] OR "low-income settings"[All Fields] OR "low income countries"[All Fields] OR "middle-income setting"[All Fields] OR "middle-income settings"[All Fields] OR "middle income countries"[All Fields] OR "transition economy"[All Fields] OR "transition economies"[All Fields] OR "economically disadvantaged"[All Fields] OR "less favored areas"[All Fields] OR "Developing World"[All Fields] OR "Developing Country"[All Fields] OR "Developing Countries"[All Fields] OR "under developed country"[All Fields] OR "under developed countries"[All Fields] OR "less developed country"[All Fields] OR "less developed countries"[All Fields] OR "less developed nation"[All Fields] OR "less developed nations"[All Fields] OR "third-world country"[All Fields] OR "third-world countries"[All Fields] OR ("africa"[MeSH Terms] OR "africa"[All Fields] OR "africa s"[All Fields] OR "africas"[All Fields]) OR ("africans"[All Fields] OR "blacks"[MeSH Terms] OR "blacks"[All Fields] OR "african"[All Fields]) OR ("asia"[MeSH Terms] OR "asia"[All Fields]) OR ("asians"[MeSH Terms] OR "asians"[All Fields] OR "asian"[All Fields]) OR "Latin America"[All Fields] OR "South America"[All Fields] OR ("afghanistan"[MeSH Terms] OR "afghanistan"[All Fields] OR "afghanistan s"[All Fields]) OR ("albania"[MeSH Terms] OR "albania"[All Fields]) OR ("algeria"[MeSH Terms] OR "algeria"[All Fields]) OR ("angola"[MeSH Terms] OR "angola"[All Fields] OR "angola s"[All Fields]) OR ("argentina"[MeSH Terms] OR "argentina"[All Fields] OR "argentina s"[All Fields] OR "argentinae"[All Fields]) OR ("armenia"[MeSH Terms] OR "armenia"[All Fields]) OR ("azerbaijan"[MeSH Terms] OR "azerbaijan"[All Fields]) OR ("bangladesh"[MeSH Terms] OR "bangladesh"[All Fields] OR "bangladesh s"[All Fields]) OR ("republic of belarus"[MeSH Terms] OR ("republic"[All Fields] AND "belarus"[All Fields]) OR "republic of belarus"[All Fields] OR "belarus"[All Fields]) OR ("belize"[MeSH Terms] OR "belize"[All Fields]) OR ("benin"[MeSH Terms] OR "benin"[All Fields] OR "benin s"[All Fields]) OR ("bhutan"[MeSH Terms] OR "bhutan"[All Fields] OR "bhutan s"[All Fields]) OR ("bolivia"[MeSH Terms] OR "bolivia"[All Fields]) OR "Bosnia and Herzegovina"[All Fields] OR ("botswana"[MeSH Terms] OR "botswana"[All Fields] OR "botswana s"[All Fields]) OR ("brazil"[MeSH Terms] OR "brazil"[All Fields] OR "brazil s"[All Fields] OR "brazils"[All Fields]) OR ("bulgaria"[MeSH Terms] OR "bulgaria"[All Fields]) OR "Burkina Faso"[All Fields] OR ("burundi"[MeSH Terms] OR "burundi"[All Fields]) OR "Cabo Verde"[All Fields] OR ("cambodia"[MeSH Terms] OR "cambodia"[All Fields] OR "cambodia s"[All Fields]) OR ("cameroon"[MeSH Terms] OR "cameroon"[All Fields] OR "cameroons"[All Fields] OR "cameroon s"[All Fields]) OR "Central African Republic"[All Fields] OR ("chad"[MeSH Terms] OR "chad"[All Fields]) OR ("china"[MeSH Terms] OR "china"[All Fields] OR "china s"[All Fields] OR "chinas"[All Fields]) OR ("colombia"[MeSH Terms] OR "colombia"[All Fields] OR "colombia s"[All Fields]) OR ("comoros"[MeSH Terms] OR "comoros"[All Fields] OR "comoro"[All Fields]) OR ("congo"[MeSH Terms] OR "congo"[All Fields]) OR "Costa Rica"[All Fields] OR "Cote d'Ivoire"[All Fields] OR ("cuba"[MeSH Terms] OR "cuba"[All Fields]) OR "Democratic Republic of the Congo"[All Fields] OR ("djibouti"[MeSH Terms] OR "djibouti"[All Fields]) OR ("dominica"[MeSH Terms] OR "dominica"[All Fields]) OR "Dominican Republic"[All Fields] OR "DR Congo"[All Fields] OR ("ecuador"[MeSH Terms] OR "ecuador"[All Fields] OR "ecuador s"[All Fields]) OR ("egypt"[MeSH Terms] OR "egypt"[All Fields] OR "egypt s"[All Fields]) OR "El Salvador"[All Fields] OR "Equatorial Guinea"[All Fields] OR ("eritrea"[MeSH Terms] OR "eritrea"[All Fields]) OR ("eswatini"[MeSH Terms] OR "eswatini"[All Fields]) OR ("ethiopia"[MeSH Terms] OR "ethiopia"[All Fields] OR "ethiopia s"[All Fields]) OR ("fiji"[MeSH Terms] OR "fiji"[All Fields]) OR ("gabon"[MeSH Terms] OR "gabon"[All Fields]) OR ("gambia"[MeSH Terms] OR "gambia"[All Fields] OR "gambia s"[All Fields]) OR ("georgia"[MeSH Terms] OR "georgia"[All Fields] OR "georgia republic"[MeSH Terms] OR ("georgia"[All Fields] AND "republic"[All Fields]) OR "georgia republic"[All Fields] OR "georgia s"[All Fields]) OR ("ghana"[MeSH Terms] OR "ghana"[All Fields] OR "ghana s"[All Fields]) OR ("grenada"[MeSH Terms] OR "grenada"[All Fields]) OR ("guatemala"[MeSH Terms] OR "guatemala"[All Fields] OR "guatemala s"[All Fields]) OR ("guinea"[MeSH Terms] OR "guinea"[All Fields] OR "guinea s"[All Fields] OR "guineas"[All Fields]) OR ("guinea bissau"[MeSH Terms] OR "guinea bissau"[All Fields] OR ("guinea"[All Fields] AND "bissau"[All Fields]) OR "guinea bissau"[All Fields]) OR ("guyana"[MeSH Terms] OR "guyana"[All Fields]) OR ("haiti"[MeSH Terms] OR "haiti"[All Fields] OR "haiti s"[All Fields]) OR ("honduras"[MeSH Terms] OR "honduras"[All Fields]) OR ("india"[MeSH Terms] OR "india"[All Fields] OR "india s"[All Fields] OR "indias"[All Fields]) OR ("indonesia"[MeSH Terms] OR "indonesia"[All Fields] OR "indonesia s"[All Fields] OR "indonesias"[All Fields]) OR ("iran"[MeSH Terms] OR "iran"[All Fields]) OR ("iraq"[MeSH Terms] OR "iraq"[All Fields]) OR ("jamaica"[MeSH Terms] OR "jamaica"[All Fields] OR "jamaica s"[All Fields]) OR ("jordan"[MeSH Terms] OR "jordan"[All Fields]) OR ("kazakhstan"[MeSH Terms] OR "kazakhstan"[All Fields] OR "kazakhstan s"[All Fields]) OR ("kenya"[MeSH Terms] OR "kenya"[All Fields] OR "kenya s"[All Fields]) OR ("micronesia"[MeSH Terms] OR "micronesia"[All Fields] OR "kiribati"[All Fields]) OR "Democratic People's Republic of Korea"[All Fields] OR "North Korea"[All Fields] OR ("kyrgyzstan"[MeSH Terms] OR "kyrgyzstan"[All Fields]) OR ("laos"[MeSH Terms] OR "laos"[All Fields]) OR ("lebanon"[MeSH Terms] OR "lebanon"[All Fields] OR "lebanon s"[All Fields]) OR ("lesotho"[MeSH Terms] OR "lesotho"[All Fields]) OR ("liberia"[MeSH Terms] OR "liberia"[All Fields] OR "liberia s"[All Fields]) OR ("libya"[MeSH Terms] OR "libya"[All Fields]) OR ("republic of north macedonia"[MeSH Terms] OR ("republic"[All Fields] AND "north"[All Fields] AND "macedonia"[All Fields]) OR "republic of north macedonia"[All Fields] OR "macedonia"[All Fields]) OR ("madagascar"[MeSH Terms] OR "madagascar"[All Fields] OR "madagascar s"[All Fields]) OR ("malawi"[MeSH Terms] OR "malawi"[All Fields] OR "malawi s"[All Fields]) OR ("malaysia"[MeSH Terms] OR "malaysia"[All Fields] OR "malaysia s"[All Fields]) OR ("indian ocean islands"[MeSH Terms] OR ("indian"[All Fields] AND "ocean"[All Fields] AND "islands"[All Fields]) OR "indian ocean islands"[All Fields] OR "maldives"[All Fields] OR "maldive"[All Fields]) OR ("mali"[MeSH Terms] OR "mali"[All Fields]) OR "Marshall Islands"[All Fields] OR ("mauritania"[MeSH Terms] OR "mauritania"[All Fields]) OR ("mexico"[MeSH Terms] OR "mexico"[All Fields] OR "mexico s"[All Fields] OR "mexicos"[All Fields]) OR ("micronesia"[MeSH Terms] OR "micronesia"[All Fields]) OR "Moldova"[All Fields] OR ("mongolia"[MeSH Terms] OR "mongolia"[All Fields] OR "mongolia s"[All Fields]) OR ("montenegro"[MeSH Terms] OR "montenegro"[All Fields]) OR ("morocco"[MeSH Terms] OR "morocco"[All Fields]) OR ("mozambique"[MeSH Terms] OR "mozambique"[All Fields] OR "mozambique s"[All Fields]) OR ("myanmar"[MeSH Terms] OR "myanmar"[All Fields] OR "myanmar s"[All Fields] OR "myanmars"[All Fields]) OR ("namibia"[MeSH Terms] OR "namibia"[All Fields]) OR ("nepal"[MeSH Terms] OR "nepal"[All Fields] OR "nepal s"[All Fields]) OR ("nicaragua"[MeSH Terms] OR "nicaragua"[All Fields] OR "nicaragua s"[All Fields]) OR ("niger"[MeSH Terms] OR "niger"[All Fields]) OR ("nigeria"[MeSH Terms] OR "nigeria"[All Fields] OR "nigeria s"[All Fields]) OR ("polynesia"[MeSH Terms] OR "polynesia"[All Fields] OR "niue"[All Fields]) OR ("pakistan"[MeSH Terms] OR "pakistan"[All Fields] OR "pakistan s"[All Fields]) OR "Papua New Guinea"[All Fields] OR ("paraguai"[All Fields] OR "paraguay"[MeSH Terms] OR "paraguay"[All Fields]) OR ("peru"[MeSH Terms] OR "peru"[All Fields]) OR ("philippine"[All Fields] OR "philippines"[MeSH Terms] OR "philippines"[All Fields]) OR ("russia"[MeSH Terms] OR "russia"[All Fields] OR "russia s"[All Fields] OR "russias"[All Fields]) OR ("rwanda"[MeSH Terms] OR "rwanda"[All Fields] OR "rwanda s"[All Fields]) OR "Saint Lucia"[All Fields] OR "Saint Vincent and the Grenadines"[All Fields] OR ("samoa"[MeSH Terms] OR "samoa"[All Fields] OR "samoas"[All Fields]) OR "Sao Tome And Principe"[All Fields] OR ("senegal"[MeSH Terms] OR "senegal"[All Fields] OR "senegal s"[All Fields]) OR ("serbia"[MeSH Terms] OR "serbia"[All Fields]) OR "Sierra Leone"[All Fields] OR "Solomon Islands"[All Fields] OR ("somalia"[MeSH Terms] OR "somalia"[All Fields]) OR "South Africa"[All Fields] OR "South Sudan"[All Fields] OR "Sri Lanka"[All Fields] OR ("sudan"[MeSH Terms] OR "sudan"[All Fields] OR "sudans"[All Fields] OR "sudan s"[All Fields]) OR ("suriname"[MeSH Terms] OR "suriname"[All Fields] OR "surinam"[All Fields]) OR ("eswatini"[MeSH Terms] OR "eswatini"[All Fields] OR "swaziland"[All Fields]) OR ("syria"[MeSH Terms] OR "syria"[All Fields] OR "syria s"[All Fields]) OR ("tajikistan"[MeSH Terms] OR "tajikistan"[All Fields]) OR ("tanzania"[MeSH Terms] OR "tanzania"[All Fields] OR "tanzania s"[All Fields]) OR ("thailand"[MeSH Terms] OR "thailand"[All Fields] OR "thailand s"[All Fields]) OR ("timor leste"[MeSH Terms] OR "timor leste"[All Fields] OR ("timor"[All Fields] AND "leste"[All Fields]) OR "timor leste"[All Fields]) OR ("togo"[MeSH Terms] OR "togo"[All Fields]) OR ("tonga"[MeSH Terms] OR "tonga"[All Fields] OR "tonga s"[All Fields]) OR ("tunisia"[MeSH Terms] OR "tunisia"[All Fields]) OR ("turkey"[MeSH Terms] OR "turkey"[All Fields] OR "turkey s"[All Fields] OR "turkeys"[MeSH Terms] OR "turkeys"[All Fields]) OR ("turkmenistan"[MeSH Terms] OR "turkmenistan"[All Fields]) OR ("micronesia"[MeSH Terms] OR "micronesia"[All Fields] OR "tuvalu"[All Fields]) OR ("uganda"[MeSH Terms] OR "uganda"[All Fields] OR "uganda s"[All Fields]) OR ("ukraine"[MeSH Terms] OR "ukraine"[All Fields] OR "ukraine s"[All Fields]) OR ("uzbekistan"[MeSH Terms] OR "uzbekistan"[All Fields]) OR ("vanuatu"[MeSH Terms] OR "vanuatu"[All Fields]) OR ("venezuela"[MeSH Terms] OR "venezuela"[All Fields] OR "venezuela s"[All Fields]) OR ("vietnam"[MeSH Terms] OR "vietnam"[All Fields] OR "vietnam s"[All Fields]) OR ("yemen"[MeSH Terms] OR "yemen"[All Fields]) OR ("zambia"[MeSH Terms] OR "zambia"[All Fields] OR "zambia s"[All Fields]) OR ("zimbabwe"[MeSH Terms] OR "zimbabwe"[All Fields] OR "zimbabwe s"[All Fields]) |
| #2 | "COVID-19"[All Fields] OR "covid*"[All Fields] OR "SARSCoV-2"[All Fields] OR "coronavirus*"[All Fields] OR "2019-nCoV disease"[All Fields] OR "betacoronavirus"[All Fields] OR "nCoV"[All Fields] OR "severe acute respiratory syndrome coronavirus 2"[All Fields] |
| #3 | "malaria*"[All Fields] OR "chills"[MeSH Terms] OR "chills"[All Fields] OR "ague"[All Fields] OR "jungle fever"[All Fields] OR "wetlands"[MeSH Terms] OR "wetlands"[All Fields] OR "marsh"[All Fields] OR "marshes"[All Fields] OR "swamp fever"[All Fields] OR "miasma"[All Fields] OR "miasmas"[All Fields] OR "malaria"[MeSH Terms] OR "malaria"[All Fields] OR "paludism"[All Fields] |
| #4 | #1 AND #2 AND #3 [Filters: from 2020 – 2021] |
