# Supplementary material for: Barriers against and strategies for malaria control during the COVID-19 pandemic in low- and middle-income countries: a systematic review
Source: Malar J. 2023 Feb 3;22:41. doi: 10.1186/s12936-023-04452-2 (PMC9896667; doi:10.1186/s12936-023-04452-2)
Supplement: Supplementary file 2 — Additional file 2: Quality appraisal results. [file 12936_2023_4452_MOESM2_ESM.docx]

**Additional File 2. Quality Appraisal Results**

| **MMAT**^a^ **(n=18)** | | | | | | | | | | | | |
| --- | --- | --- | --- | --- | --- | --- | --- | --- | --- | --- | --- | --- |
| Qualitative (n=2) | S1  Research questions | S2  Data appropriate-ness | | 1.1  Approach rationale | | 1.2  Data appropriate-ness | | 1.3  Data Derived | | 1.4  Result interpretation | | 1.5  Coherence |
| Aïkpon et al.(2020) | 1 | 1 | | 1 | | 1 | | 1 | | 1 | | 1 |
| Feldman et al.(2021) | 1 | 1 | | 1 | | 0 | | 1 | | 1 | | 1 |
| Quantitative  non-randomized (n=7) | S1  Research questions | S2  Data appropriate-ness | | 3.1  Recruitment | | 3.2  Appropriatemeasures | | 3.3  Complete outcomes | | 3.4  Confounders | | 3.5  Intended intervention |
| Buonsenso et al. (2020a) | 1 | 1 | | 1 | | 1 | | 1 | | 0 | | 1 |
| Engoba et al. (2021) | 1 | 1 | | 1 | | 1 | | 1 | | 0 | | 1 |
| Hategeka et al. (2021) | 1 | 1 | | 0 | | 1 | | 1 | | 0 | | 1 |
| Heuschen et al. (2021) | 1 | 1 | | 1 | | 1 | | 1 | | 0 | | 1 |
| Mbunge et al. (2021) | 1 | 1 | | 1 | | 0 | | 1 | | 0 | | 1 |
| Namuganga et al. (2021) | 1 | 1 | | 1 | | 1 | | 1 | | 1 | | 1 |
| Thapa et al. (2020) | 1 | 1 | | 1 | | 1 | | 1 | | 0 | | 0 |
| Quantitative descriptive (n=6) | S1 Research questions | S2  Data appropriate-ness | | 4.1  Relevant sampling | | 4.2  Representative sampling | | 4.3  Appropriatemeasures | | 4.4  nonresponse bias | | 4.5  Appropriateanalysis |
| Buonsenso et al. (2020b) | 1 | 1 | | 1 | | 1 | | 1 | | 0 | | 1 |
| Enyindah et al. (2021)^b^ | 1 | 0 | | - | | - | | - | | - | | - |
| Gavi et al. (2021) | 1 | 1 | | 1 | | 1 | | 1 | | 0 | | 1 |
| Ilesanmi et al. (2021) | 1 | 1 | | 1 | | 0 | | 1 | | 1 | | 1 |
| Seboka et al. (2021) | 1 | 1 | | 0 | | 0 | | 1 | | 0 | | 1 |
| Suiyanka et al. (2021) | 1 | 1 | | 1 | | 1 | | 1 | | 1 | | 1 |
| Mixed methods (n=3) | S1  Research questions | S2  Data appropriate-ness | | 5.1  Approach rationale | | 5.2  Component integration | | 5.3  Result interpretation | | 5.4  Inconsistency addressed | | 5.5  Methodological quality |
| Afai et al. (2021) | 1 | 1 | | 1 | | 1 | | 1 | | 1 | | 0 |
| Hakizimana et al. (2021) | 1 | 1 | | 1 | | 1 | | 1 | | 1 | | 1 |
| Ward et al. (2021) | 1 | 1 | | 0 | | 1 | | 1 | | 1 | | 1 |
| **AACODS**^a^ **(n=12)** | | | | | | | | | | | | |
|  | Q.1  Authority | | Q.2  Accuracy | | Q.3  Coverage | | Q.4  Objectivity | | Q.5  Date | | Q.6  Significance | |
| AMP (2020) | 1 | | 0 | | 1 | | 0 | | 1 | | 0 | |
| PMI (2021) | 1 | | 0 | | 1 | | 0 | | 1 | | 0 | |
| The Commonwealth (2021) | 1 | | 1 | | 1 | | 1 | | 0 | | 1 | |
| The Global Fund (2020) | 1 | | 1 | | 0 | | 1 | | 1 | | 1 | |
| The Global Fund (2021a) | 1 | | 0 | | 1 | | 0 | | 1 | | 0 | |
| The Global Fund (2021b) | 1 | | 1 | | 1 | | 1 | | 1 | | 1 | |
| WHO (2020a) | 1 | | 1 | | 1 | | 1 | | 1 | | 1 | |
| WHO (2020b) | 1 | | 1 | | 1 | | 1 | | 1 | | 1 | |
| WHO (2021a) | 1 | | 1 | | 1 | | 1 | | 1 | | 1 | |
| WHO (2021b) | 1 | | 1 | | 1 | | 1 | | 1 | | 1 | |
| WHO (2021c) | 1 | | 1 | | 1 | | 0 | | 1 | | 1 | |
| Wu et al. (2021) | 1 | | 1 | | 1 | | 1 | | 1 | | 1 | |

^a^ Yes = 1, No = 0

^b^ For MMAT, any zeros(0) in the two screening questions (S1 and S2) indicate that further appraisal of other questionnaires is not feasible. This study was excluded for being non-empirical.
